# Response of soil organic carbon to vegetation degradation along a moisture gradient in a wet meadow on the Qinghai–Tibet Plateau

**DOI:** 10.1002/ece3.4656

**Published:** 2018-11-06

**Authors:** Abdul‐Rauf Malimanga Alhassan, Weiwei Ma, Guang Li, Zhirong Jiang, Jiangqi Wu, Guopeng Chen

**Affiliations:** ^1^ College of Forestry Gansu Agricultural University Lanzhou China; ^2^ Ministry of Food and Agriculture Tamale Ghana; ^3^ Institute of Atmospheric Physics Chinese Academy of Sciences Beijing China

**Keywords:** climate change, ecosystems, global carbon cycle, grasslands, wet meadows, wetlands

## Abstract

The study was conducted during the growing seasons of 2013, 2014, and 2015 in the wet meadows on the eastern Qinghai–Tibet plateau (QTP) in the Gansu Gahai Wetland Nature Reserve to determine the dynamics of soil organic carbon (SOC) as affected by vegetation degradation along a moisture gradient and to assess its relationship with other soil properties and biomass yield. Hence, we measured SOC at depths of 0–10, 10–20, and 20–40 cm under the influence of four categories of vegetation degradation (healthy vegetation [HV], slightly degraded [SD], moderately degraded [MD], and heavily degraded [HD]). Our results showed that SOC decreased with increased degree of vegetation degradation. Average SOC content ranged between 36.18 ± 4.06 g/kg in HD and 69.86 ± 21.78 g/kg in HV. Compared with HV, SOC content reduced by 30.49%, 42.22%, and 48.22% in SD, MD, and HD, respectively. SOC significantly correlated positively with soil water content, aboveground biomass, and belowground biomass, but significantly correlated negatively with soil temperature and bulk density (*p* < 0.05). Highly Significant positive correlations were also found between SOC and total nitrogen (*p* = 0.0036), total phosphorus (*p* = 0.0006) and total potassium (*p* < 0.0001). Our study suggests that severe vegetation and moisture loss led to approximately 50% loss in SOC content in the wet meadows, implying that under climate warming, vegetation and soil moisture loss will dramatically destabilize carbon sink capacities of wetlands. We therefore suggest wetland hydrological management, restoration of vegetation, plant species protection, regulation of grazing activities, and other anthropogenic activities to stabilize carbon sink capacities of wetlands.

## 1 INTRODUCTION

Soil organic carbon (SOC) constitutes over 60% of total soil carbon (Lal, 2004) and significantly influences soil properties, plant growth, and environmental quality (Sharma et al., 2008). Loss of SOC can decrease availability of other soil nutrients, and destroy soil structure (Bronick & Lal, 2005). Investigations of SOC changes are therefore critical in global carbon cycle analyses. Higher biota productivity, higher soil water conditions, and slower organic matter decomposition in wetlands result in improved carbon sequestration (Whitaker et al., 2015). Anaerobic conditions in wetlands inhibit CO_2_ production (Chmura, Kellman, & Guntenspergen, 2011) thereby reducing carbon output to the atmosphere and this may result in net carbon sequestration. These conditions have resulted in significant carbon stocks in wetland ecosystems which constitute 20%–30% of the earth's total carbon pool (Bridgham, Megonigal, Keller, Bliss, & Trettin, 2006) and 45%–70% of terrestrial organic carbon (Mitra et al. 2005).

The Gahai Lake, located on the Qinghai–Tibet Plateau (QTP) in north‐western China, is the largest geomorphologic unit on the Eurasian continent (Wang, Wu, Tian, Niu, & Tan, 2012) and is endowed with abundance of large areas of natural wetlands, grasslands, and alpine lakes (Li & Zhou, 1998). It has been estimated that the QTP stores 23.44% of the total SOC in China, representing 2.50% of the world SOC storage (Wang, Qian, Cheng, & Lai, 2002). It has however been reported that wetland areas on the QTP are degrading and declining due to global warming and human activities (Wang et al., 2008; Zheng, Zhang, Niu, & Gong, 2012). The wet meadows on the eastern margin of the QTP serve both as wetlands and grasslands and have undergone marked shrinkage due to vegetation loss and declining soil moisture as consequences of global warming and human activities (Gao, Zhang, Lei, & Wang, 2014). Russi et al. (2013) reported that there is clear neglect of this wetland type, globally in the compilation of wetland inventories. Furthermore, the impacts of climate change on this wetland type is often overlooked even though they are widely distributed, differentiated geographically but also exhibit similar features in vegetation and hydrology on a global level, hence they are important ecosystems for climate change studies (Joyce, Simpson, & Casanova, 2016). On the eastern edge of the QTP, there was an overt loss in vegetation along a moisture gradient in the wet meadows yet no study has been carried out on the response of SOC along these gradients. Although a number of studies have been conducted on SOC dynamics on the QTP (Liu et al., 2014; Liu, Chen, Qin, et al., 2012; Wang et al., 2002; Yang, Fang, Tang, Ji, & Zhu, 2008), these studies compared SOC storage among different ecosystems at wide spatial scales with little information and emphasis on environmental changes such as vegetation and moisture variations in similar ecosystems at the landscape and field levels. Vegetation and moisture changes are important criteria for assessing wetland environmental changes. Gao, Li, Cheung, and Yang (2013), hypothesized that there are differences in the effectiveness of indicators for assessment of wetland degradation levels, and concluded that vegetation composition and amount, as well as moisture levels are the most effective indicators for assessing ecological health of wetland ecosystems on the QTP. Furthermore, most of these studies are often relatively short‐term (maximum of a year), with non‐continuous sampling. Therefore, extensive field research is direly needed. Norton, Olsen, Jungst, Legg, and Horwath (2014) conducted a quantitative study on SOC and total nitrogen (TN) storage in alluvial wet meadows on the west slope of central Sierra Nevada, highlighting SOC changes along the hydrological gradient. However, such study is scarce in China.

This study sought to investigate the change patterns of SOC and its relationship with soil chemical properties as well as soil water content (SWC), soil temperature (ST), aboveground biomass (AGB), belowground biomass (BGB), and bulk density (BD) in wet meadow wetlands along a moisture and vegetation degradation gradient. The study hypothesizes that vegetation and moisture loss influence SOC distribution in wet meadow ecosystems due to their influence on biomass productivity and other soil properties. This study provides basic data for establishing and updating global soil carbon inventory, tracking long term SOC change rates, and for realistic prediction of ecosystem carbon storage capacity. It also provides data for development of mitigation measures and policy guidelines for effective wetland management and restoration on the QTP and other plateau ecosystems globally.

## 2 MATERIALS AND METHODS

### 2.1 Study area

**Figure 1:**
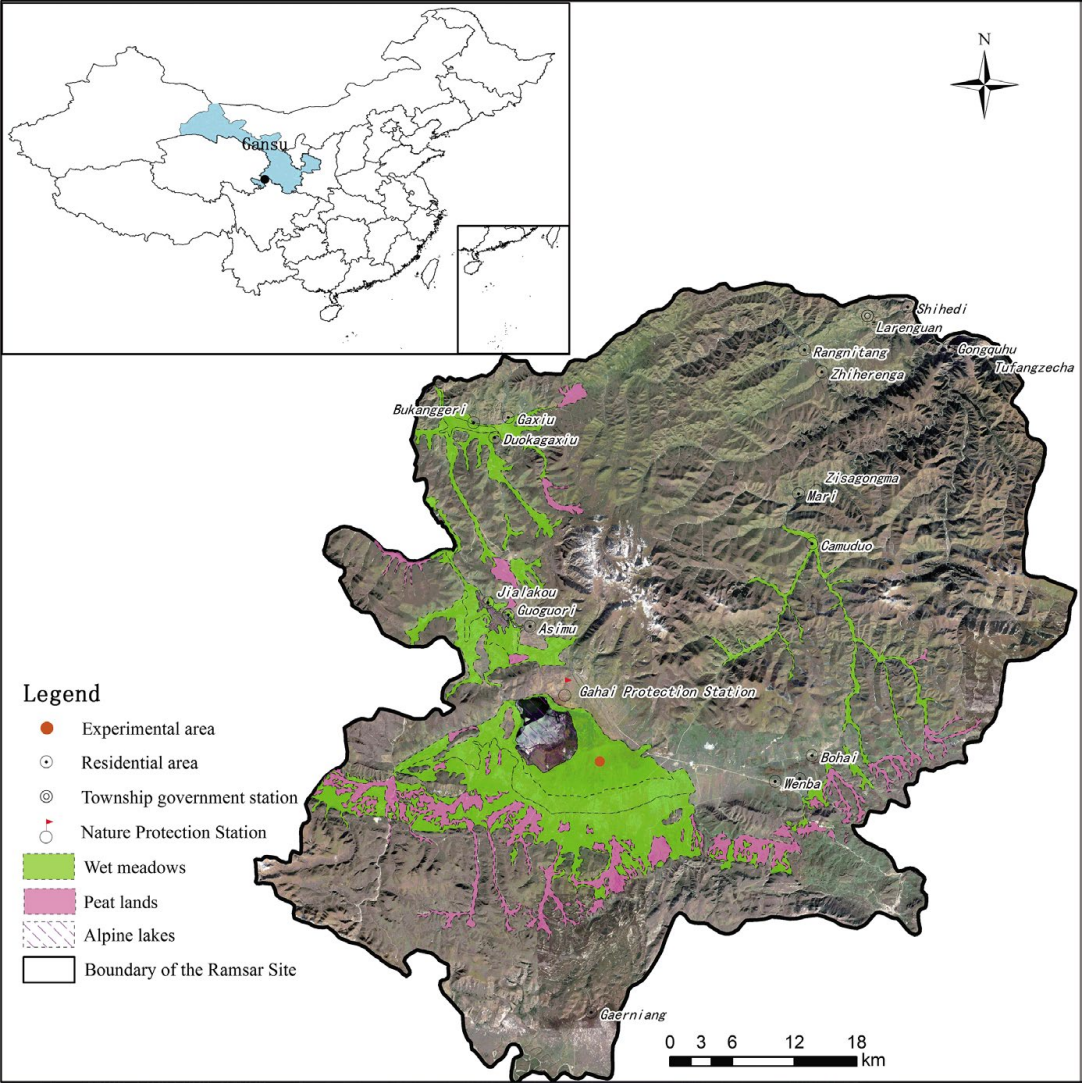
Distribution of wet meadows in the Gansu Gahai Wetland Nature Reserve

**Figure 2:**
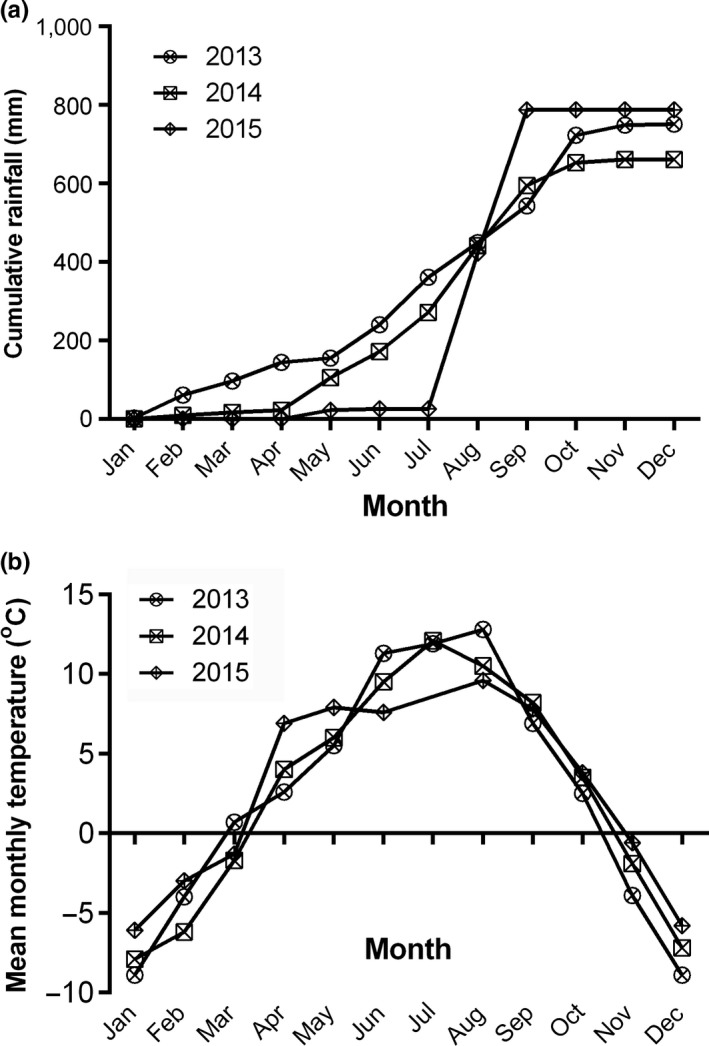
Cumulative rainfall (a) and mean monthly temperature (b) of the weather station of Gansu Gahai Wetland Protection Station for 2013, 2014, and 2015

This study was conducted in the wet meadows in Gansu Gahai Wetland Nature Reserve (34°16'N, 102°26'E) on the eastern QTP (Figure 1), during the growing seasons of 2013, 2014, and 2015. The dominant ecosystem vegetation type is wet meadows which cover an area of 4.07 × 10^4^ ha in the reserve, representing 70% of the area of ecosystems on the entire reserve. The nature reserve is located in the south of Gansu province of China on an altitude between 3,430 and 4,300 m above sea level (asl), and is characterized by a cool continental climate according to the köppen climate classification and forms an important part of the QTP. The nature reserve was listed as one of the Ramsar wetlands of international importance (Sun et al., 2014). Annual cumulative rainfall recorded in the weather station located at the nature reserve in 2013, 2014, and 2015 were 751.11, 661.40, and 787.95 mm, respectively (Figure 2a). The growing season starts in May and ends in September within which period approximately 80% of rainfall occurs and this period recorded relatively warmer monthly average temperatures ranging between 5.50°C and 12.80°C (Figure 2b). Annual average air temperature for the 3‐year study period was 2.40°C with minimum daily air temperature of −26.20°C in February, 2014 and maximum temperature of 26.20°C recorded in September, 2013 (Table 1). Several wet meadows, found close to the Gahai Lake exhibit wet conditions in the growing season but gradually reduce in moisture toward the winter season. The wet meadows also contain nutritious grass species thereby serving as both wetlands and grasslands which are usually grazed in the non‐growing season. These grazing activity, coupled with warmer climate over the years (Gao & Li, 2016), have exposed the ecosystems to degradation of various levels.

**Table 1:** Monthly mean, maximum and minimum temperatures in the Gansu Gahai wetland Protection Station in 2013, 2014, and 2015

Month	Temperature (^o^C)								
	2013			2014			2015		
	Mean	Maximum	Minimum	Mean	Maximum	Minimum	Mean	Maximum	Minimum
Jan	−8.9	11.3	−23.9	−7.9	10.2	−22.1	−6.1	12.3	−24.1
Feb	−4	12.3	−19.5	−6.2	9.6	−26.2	−3.0	12.7	−14.6
Mar	0.7	14.8	−15	−1.7	14.1	−14.4	−1.3	19.5	−12.2
Apr	2.6	17.4	−9.4	4	16.8	−4.1	6.9	19.7	−7.6
May	5.5	22.6	−3.8	6	18.7	−8	7.9	21.4	−5.6
Jun	11.3	22.6	−3.4	9.5	23.7	0.9	7.6	21.8	−0.1
Jul	11.9	24.9	1.8	12.1	24.8	1.4	[Link]	[Link]	[Link]
Aug	12.8	22.8	1.1	10.5	22.3	−0.5	9.6	22.3	−0.5
Sep	6.9	26.2	−4.7	8.2	21.3	−2	7.8	20.5	−1.2
Oct	2.5	20.1	−9.1	3.5	17.9	−6.1	3.8	16	−7.3
Nov	−3.9	21.4	−18.1	−1.9	13.9	−13.3	−0.6	13.3	−17
Dec	−8.9	11.3	−24.1	−7.2	9.6	−20.1	−5.8	8.4	−23.2

Data for July, 2015 were not obtained due to malfunction of the weather station.

### 2.2 Experimental design

Four treatments with three replications were laid out along a moisture gradient which also showed a clear vegetation degradation gradient (Table 2). A previous survey in this area (Ma, Wang, Li, & Shi, 2015) categorized the wet meadows into four stages of ecological health according to vegetation coverage and composition as: Intact or healthy vegetation, slightly degraded, moderately degraded, and severely or heavily degraded. This classification is in line with classification of degraded wetlands on the QTP by Gao, Schumann, Zeng, and Chen (2011). In each degradation category, three plots, measuring 10 m × 10 m were demarcated for soil sampling while a small area (0.5 m × 0.5 m) within each of the 10 m × 10 m plots were demarcated for biomass sampling. The three plots sampled within each degradation class represented three replications for soil and biomass. The treatments used for this study were coded as: Healthy vegetation (HV); slightly degraded (SD); moderately degraded (MD); and heavily degraded (HD). The dominant plant species found in HV were *Kobresia tibetica*,* Potentilla anserine* L., and *Poa annua* with vegetation cover of 96% and average plant height of 16.31 cm. The SD treatment contained dominant plant species such as *Carex* sp., and *Oxytropis* sp., with vegetation cover of 86% and average plant height of 13.33 cm. Moderately degraded also contained *Artemisia sacrorum* var., *messerschmidtiana* and *Kobresia capilifolia* with 45% vegetation coverage and average plant height of 7.61 cm while HD was severely degraded with only little of *Polygonum viviparum* species found.

**Table 2:** Average values of soil properties (0–40 cm depth) and vegetation status (0–30 cm) in alpine meadows of different degradation stages

Treatment	TN	TP	TK	BD	SWC	ST	AGB	BGB
	g/kg			g/cm^3^	%	^o^C	g/m^2^	
HV	2.38 ± 0.78a	1.61 ± 0.16a	6.14 ± 0.24a	0.36 ± 0.02c	50.69 ± 8.30a	12.64 ± 3.10a	378.40 ± 12.21a	4,583.16 ± 410.86a
SD	2.03 ± 0.63a	1.41 ± 0.23a	6.16 ± 0.21a	0.39 ± 0.02c	38.41 ± 4.75b	14.05 ± 3.12a	308.07 ± 16.91b	3,008.63 ± 262.35b
MD	1.87 ± 0.51b	1.21 ± 0.02ab	5.78 ± 0.06b	0.56 ± 0.03b	23.80 ± 1.60c	15.97 ± 3.73b	261.22 ± 6.82c	1,290.73 ± 205.41c
HD	1.76 ± 0.56b	1.24 ± 0.09b	5.75 ± 0.11b	0.61 ± 0.05a	21.53 ± 1.22c	17.14 ± 4.14b		

Note

Different letters in the same column indicate significant difference at *p* < 0.05.

AGB: aboveground biomass; BD: bulk density; BGB: belowground biomass; HD: heavily degraded; HV: healthy vegetation, MD: moderately degraded; SD: slightly degraded; ST: soil temperature; SWC: soil water content; TK: total potassium; TN: total nitrogen; TP: total phosphorus.

### 2.3 Soil sampling and analysis

Five soil samples were taken with a soil corer of 4.0 cm in diameter, along a *Z*‐plane at depths of 0–10, 10–20, and 20–40 cm in every plot. Soil samples were taken once a month in all the four vegetation classes with three replicates for 5‐months between May and September for each of the study years. Samples within the same depth were mixed thoroughly. The composite samples were then air dried and sieved through a 2 mm sieve to remove plant debris. A subsample was further sieved with a 0.25 mm sieve and analyzed for SOC, TN, total phosphorus (TP), and total potassium (TK). Soil organic carbon was analyzed using the Walkley‐Black dichromate oxidation method (Nelson & Sommers, 1982) by oxidizing organic matter using a mixture of potassium dichromate (K_2_Cr_2_O_7_) and sulfuric acid (H_2_SO_4_) and titrated against ferrous sulfate (FeSO_4_). About 0.1 g of air dried soil sample was extracted with 7.5 ml of 0.4 M of K_2_Cr_2_O_7_ and 7.5 ml of concentrated H_2_SO_4_ at 150°C for 30 min. Analysis of TN was carried out by the semi micro‐Kjeldahl method (ISSCAS, 1978). We measured TP using the molybdenum–antimony anti‐spectrophoto‐metric method with H_2_SO_4_–HClO_4_ as the digester (Zhou, 1996), whiles TK was measured by flame photometry with HF–HClO_4_ as the digester (Liu, Chen, Hu, & Sha, 2012).

Soil water content (%) was determined gravimetrically at depths of 0–10, 10–20, and 20–40 cm using the difference between the weight of moist soil samples and the dry weight after oven drying at 105°C for 24 hr (Liu et al., 2014). Soil bulk density (g/cm^3^), were also determined at depths of 0–10, 10–20, and 20–40 cm using the bevelled stainless steel ring method (Carter & Gregorich, 1993) with a cutting ring of 10 cm in diameter. A temperature sensor (JM624; Jinming Instrument Co., Tianjing, China) was used to measure ST at 5, 10, and 20 cm depths concurrently with soil sampling. Soil chemical, physical properties as well as hydrothermal conditions of the sites are shown in Table 2.

### 2.4 Biomass sampling

Biomass assessment was conducted during the growing seasons for the 3‐year experimental period. Above and below ground plant materials from three randomly selected quadrats (0.5 m × 0.5 m) within HV, SD, and MD plots were collected and oven dried at 80°C to constant weight. All above ground plant parts within the demarcated plots were harvested by cutting near the soil surface for AGB measurement. Below ground biomass was measured after collecting samples from 0–10 cm, 10–20 cm, and 20–30 cm with the 4 cm diameter soil corer, passed through a 2 mm sieve and then thoroughly cleaned. The total below ground biomass was obtained by summing up biomass at depths of 0–10, 10–20, and 20–30 cm. The 0–30 cm depth was selected by the assumption that root activity is limited at far deeper soil depths. Biomass assessment was not conducted for HD due to insignificant amount of vegetation found as a result of severe degradation. Biomass data for the three sampled degradation classes are shown in Table 2.

### 2.5 Statistical analysis

Statistical analyses were conducted using SPSS version 22 (IBM Corporation, Chicago, IL, USA, 2013). One‐way analysis of variance (ANOVA) was conducted to determine treatment effects on SOC at various soil depths while two‐way ANOVA was used for assessing interactions between depth and degradation stage on SOC variations. Pearson correlation analysis and linear regression were used to examine the relationship between SOC, biomass and soil physical and chemical properties. Differences in treatment means were analyzed at significance level of 95% (*p* < 0.05) using the Duncan's multiple range test.

## 3 RESULTS

### 3.1 SOC across different wetland vegetation degradation categories

**Figure 3:**
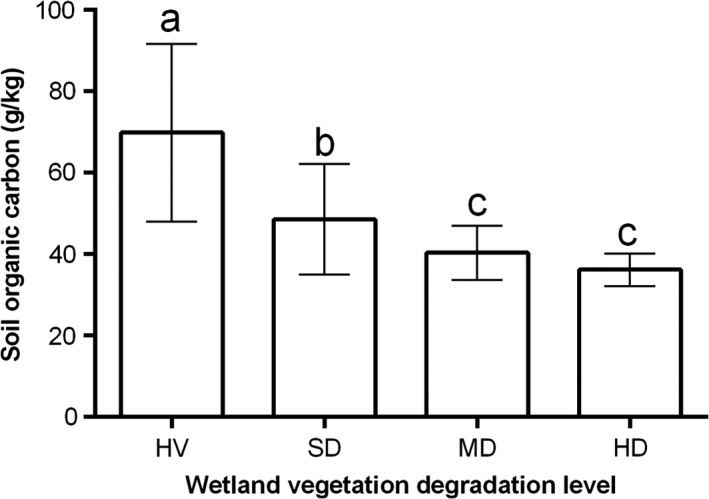
Three‐year average SOC content (0–40 cm depth) across wetlands of different vegetation degradation stages. HD: heavily degraded; HV: healthy vegetation; MD: moderately degraded; SD: slightly degraded; SOC: soil organic carbon

There were significant variations in SOC content across the vegetation and moisture gradient (Figure 3). Average SOC content found in this 3‐year study were 69.86 ± 21.78, 48.56 ± 13.54, 40.36 ± 6.70, and 36.18 ± 4.06 g/kg in HV, SD, MD, and HD, respectively. Compared with HV, SOC content reduced by 30.49%, 42.22%, and 48.22% in SD, MD, and HD wetlands, respectively. At *p* < 0.05, SOC in HV was significantly higher than that in SD, MD, and HD while that of SD was also significantly higher than MD and HD but there was no significant difference between MD and HD (Figure 3).

### 3.2 Vertical distribution of SOC

**Figure 4:**
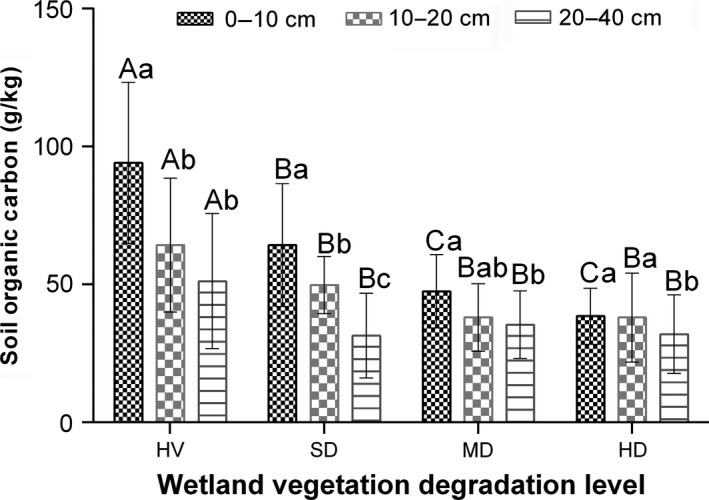
Vertical distribution of SOC content in wetlands of different vegetation degradation stages. SOC: soil organic carbon

Generally, SOC decreased with soil depth for all vegetation degradation classes with the highest SOC content found in the 0–10 cm profiles for all degradation stages (Figure 4). In HV, SD, and MD, SOC was significantly higher in the 0–10 cm depth than the other two depths sampled (10–20 and 20–40 cm), but SOC in the 0–10 cm profile in HD did not show significant difference with that of its 10–20 cm but was significantly higher than its 20–40 cm profile. Also, we found that SOC in all degradation classes in the 10–20 cm depth were significantly higher than that of the 20–40 cm depths except in SD. Interaction analyses between vegetation degradation stage and soil depth on SOC content indicated significant interactions between these two parameters (Table 3). Soil organic carbon content in HV, SD, MD, and HD were, respectively, 45.59%, 51.01%, 25.26%, and 16.88% higher in the 0–10 cm compared with the 20–40 cm depth, implying a higher proportion of SOC in the 0–10 cm of HV and SD than in MD and HD. In contrast, BD increased along soil profile by 3.58%, 4.51%, 9.64%, and 11.21% in HV, SD, MD, and HD, respectively, between the 0–10 cm and 20–40 cm soil depths (Figure 4).

**Table 3:** ANOVA table of ecosystem degradation stage and soil depth

Source	Type III sum of squares	Degree of freedom	Mean square	*F*	Sig.
Corrected model	10,913.402[Link]	11	992.127	20.213	0.000
Intercept	85,526.757	1	85,526.757	1742.501	0.000
Degradation stage	6,068.351	3	2022.784	41.212	0.000
Depth	3,358.374	2	1679.187	34.211	0.000
Degradation stage × depth	1,486.677	6	247.779	5.048	0.002
Error	1,177.986	24	49.083		
Total	97,618.145	36			
Corrected total	12,091.388	35			

*R* ^2^ = 0.903 (Adjusted *R* ^2^ = 0.858).

### 3.3 Temporal variations of SOC

**Figure 5:**
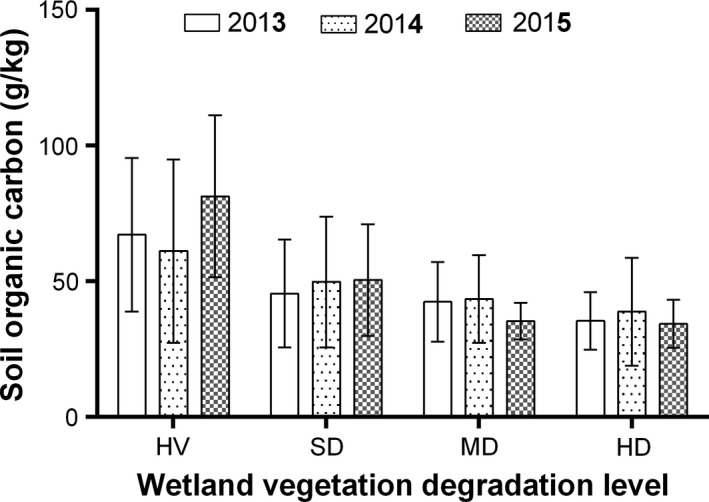
Temporal variations of SOC content in wetlands of different vegetation degradation stages. SOC: soil organic carbon

**Figure 6:**
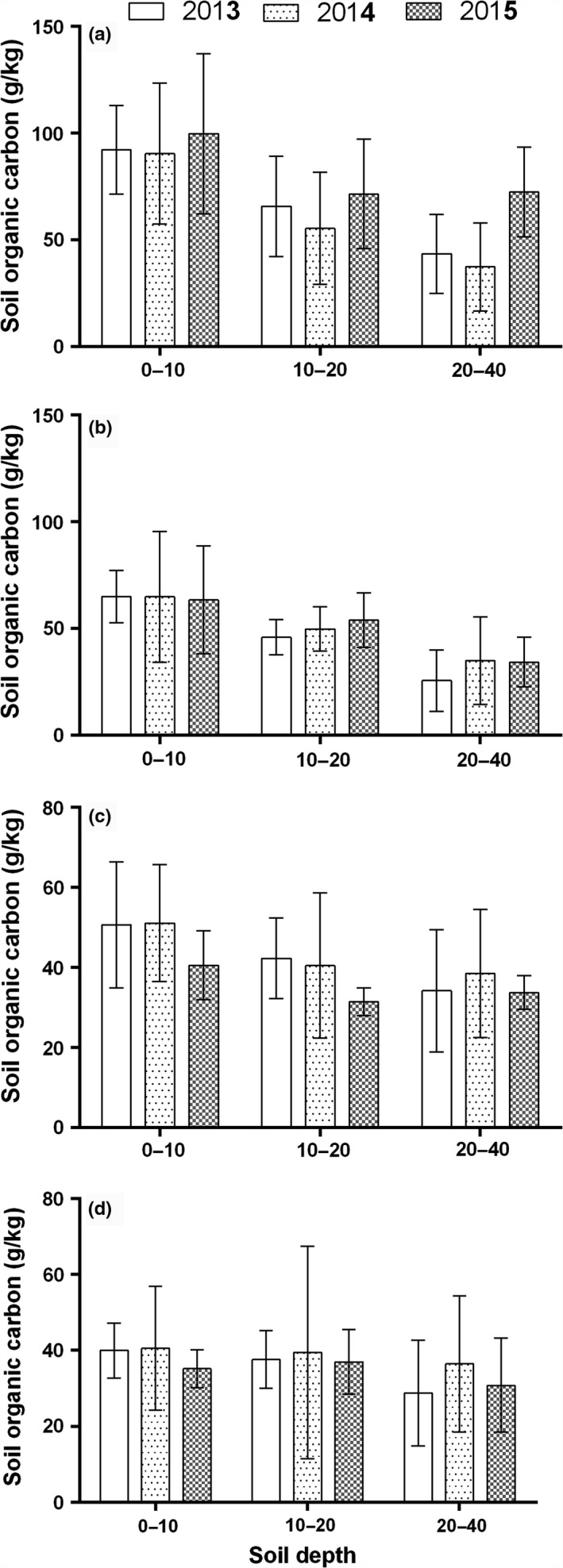
Three‐year variations of SOC content along the vertical profile in wetlands of different vegetation degradation stages. SOC: soil organic carbon

Average SOC content in HV and SD was highest in 2015 while that in MD and HD were highest in 2014. Between 2013 and 2015, average SOC content increased in HV and SD but reduced in MD and HD (Figure 5) while between 2013 and 2014, SOC in all the degradation classes increased except in HV. Averagely, vertical distribution of SOC content also showed temporal variations, with SOC content increasing in the 10–20 and 20–40 cm depths in HV and SD between 2013 and 2015 but decreased in MD and HD except in the 20–40 cm depth in HD (Figure 6).

### 3.4 Environmental controls on SOC variations

**Figure 7:**
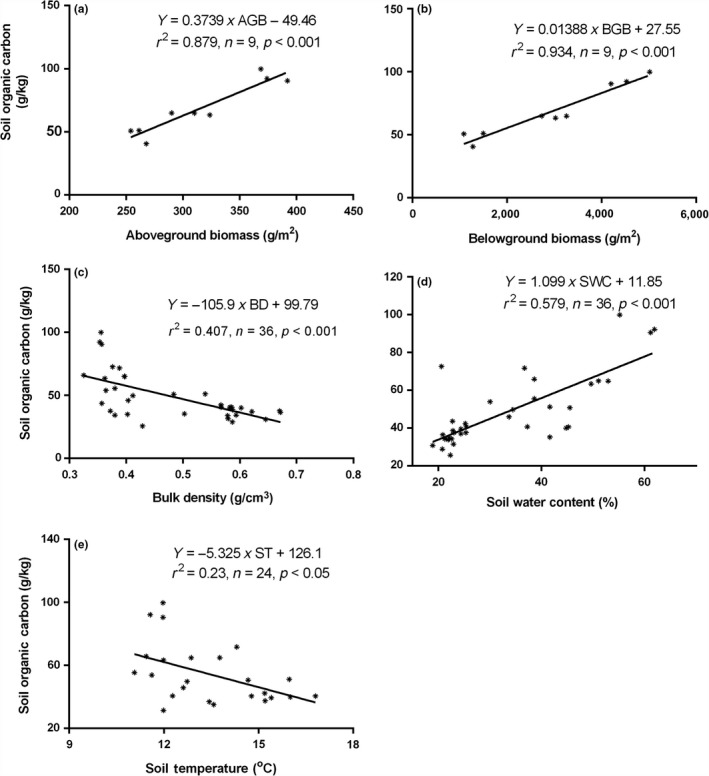
Linear regression between SOC and (a) aboveground biomass (AGB) (b) belowground biomass (BGB) (c) bulk density (BD) (d) soil water content (SWC) (e) soil temperature (ST). SOC: soil organic carbon

Linear regression analyses were conducted to examine the environmental controls on variations of SOC (Figure 7) across different levels of vegetation degradation. It revealed that AGB and BGB, when considered individually accounted for 87.93% and 93.42% of SOC variations, respectively (Figure 7a,b); BD accounted for 40.73% (Figure 7c) while SWC accounted for 57.86% of variations (Figure 7d). Soil temperature showed a significant negative relationship with SOC even though at a relatively lower strength (Figure 7e). Analyzing the controls along the soil profile, we found that the controls of SWC and BD on variations of SOC content in different wetland vegetation classes were not significant in the 20–40 cm depth (Table 4). However, within the 0–10 and 10–20 cm depths, SWC significantly correlated positively with SOC while BD correlated negatively but ST significantly correlated negatively with SOC only at the 0–10 cm depth.

**Table 4:** Correlation analyses and regression equations for bulk density, soil water content and soil temperature, against SOC at different soil depths for all treatments

Soil depth (cm)	BD (g/kg)		SWC (%)		ST (^o^C)	
	Equation	*r*	Equation	*r*	Equation	*r*
0–10	151.08–195.92*x*	−0.866**	−66.47 + 2.6*x*	0.902**	172.31–8.16*x*	−0.67*
10–20	88.24–84.39*x*	−0.840**	−5.18 + 1.78*x*	0.875**	82.24–2.60*x* [Link]	0.35 n.s.
20–40	56.10–37.15*x*	−0.384	56.38–0.87*x*	−0.088		

Note

n.s.: not statistically significant; SOC: soil organic carbon.

Soil Temperature at 0–20 cm regressed against SOC at 10–20 cm.

a Statistical significance at *p* < 0.01.

b Statistical significance at *p* < 0.05.

## 4 DISCUSSION

Soil organic carbon contents found in this study are comparable to that of the alluvial wet meadows in central Sierra Nevada in the United States (Norton et al., 2014). However, they are higher compared to studies on the Central QTP (Liu et al., 2014; Wang et al, 2012) but also within the range of those reported by Wang et al. (2002) for different grassland soils in Qinghai and Tibet. High variations in climate, soil types, vegetation composition, environmental factors, and ecosystem types on the QTP result in variations in SOC contents across the plateau. Baumann, He, Schmidt, Kühn, and Scholten (2009) expounded that spatial heterogeneity on the QTP resulted in significant disparities in SOC densities and that carbon and nitrogen stocks exhibited a decreasing trend from Southeast to Northwest due to the influence of climatic factors. The study area is known to have higher precipitation compared to the Central QTP, thus might have accounted for the relatively higher SOC in our study. Within the study period, our study area had a higher average precipitation (733.51 mm) compared to long term precipitation of 424 mm on the central QTP (Wang & Wu, 2013).

Across different vegetation degradation classes, the highest SOC content found in HV was partly due to higher AGB and BGB (Table 2). Higher biomass is often associated with increased organic matter input thus resulting in higher SOC content (Zak et al., 1994) through the process of net primary production (NPP) from AGB and carbon (C) allocation through BGB. As found in our study, AGB and BGB showed significant positive correlations with SOC content, both at *p* < 0.001 (Figure 7a,b). One other conduit for C accumulation in soil is through lignin‐derived compounds (Fissore et al., 2008). Vegetation degradation may however affect lignin decomposition by altering soil physical properties (Guo, Meng, Zhang, & Chen, 2016) leading to lower SOC in degraded wet meadows. As observed in this study, vegetation degradation significantly increased soil BD (Table 2), which showed a very strong inverse relation with SOC content (Figure 7c) at *p* < 0.001. This implied that increased bulk densities in the degraded wetlands may have contributed to the decline in SOC. Bulk density is an indicator of soil compaction and influences soil biodiversity. It has been reported that compaction could lead to reduced root penetration in soil due to increased soil strength (Kristoffersen & Riley, 2005) which reduces BGB, reduces microbial biomass, retards enzyme activities and other rhizosphere functions and thus influences chemical properties of soil (Nawaz, Bourrié, & Trolard, 2013). Higher SWC in HV wetlands might have caused a concomitant decline in ST in these wetlands (Table 2) which created favourable hydrothermal condition for higher carbon production and allocation. As shown in Figure 7d, there was a significant positive correlation between SOC and SWC (*p* < 0.001). In contrast, lower SWC and lower biomass may have induced higher ST in degraded wetlands which accounted for lower SOC contents in these wetlands since ST showed significant negative correlation with SOC (*p* < 0.05) (Figure 7e). These findings are in line with other research. Previous studies also found that SWC had a positive influence on SOC while ST exerted a negative influence on SOC (Liu et al., 2014). This could be explained by the mechanism that higher SWC enhanced nutrient supply to plants, which improved plant productivity, thus increased SOC concentration (Reichstein & Beer, 2008). Furthermore, Tang et al. (2012) explained that SWC influences soil bacterial community, and thus, it has an impact on soil carbon input. Soil water content acts as agent of bacterial motility, and influences nutrient and energy supply (Yancey, Clark, Hand, Bowlus, & Somero, 1982). Through this process, bacterial activity may increase, which may lead to higher SOC turnover rates (Cheng et al., 2013; Kuzyakov, Friedel, & Stahr, 2000). It has also been widely reported that higher temperatures result in higher SOC decomposition rates though this depends on substrate availability (Hopkins, Torn, & Trumbore, 2012), which might have increased soil respiration, hence lower SOC content in degraded wetlands. However, in our study, compared to other factors, ST showed the least correlation, with an *r* ^2^ value of 0.23 and significance at *p* < 0.05. Although soil respiration may increase due to increased ST, it has been reported that soil respiration may also adapt to temperature variations, leading to lower correlations between SOC and temperature (Zeng, Zhang, Shen, Cao, & Zhao, 2014). This implied that current changes in ST in our study area had lower effect on SOC variations compared to the effects of biomass and SWC.

**Figure 8:**
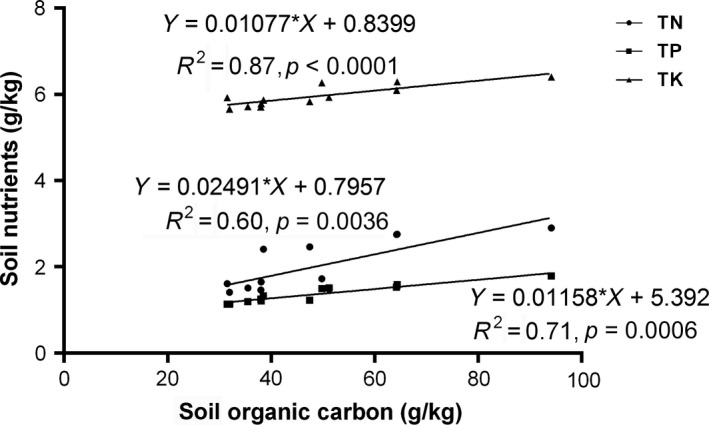
Linear regression between SOC and total nitrogen (TN), total phosphorus (TP), and total potassium (TK). SOC: soil organic carbon

As shown in Table 2, vegetation degradation and soil moisture decline have caused reductions in TN, TP, and TK in a similar fashion to SOC changes. Moges and Holden (2008) have also made similar observation where SOC and TN showed similar trends under soil degradation. This suggests that SOC and TN might be influenced by similar soil environmental changes (Wang et al., 2002) and are strongly correlated. In our study, regression analyses revealed significant positive correlations of SOC with TN (*p* = 0.0036), TP (*p* = 0.0006), and TK (*p* < 0.0001) (Figure 8). A study by Hu et al (2014) in the Loess Plateau corroborates our findings while Azam, Yousaf, Hussain, and Malik (1989) also expounded that soil organic matter is the main carrier of TN, hence, a strong correlation between TN and SOC is expected. The behavior of TP along the vegetation degradation and moisture gradient was also consistent with other studies (Cheng & An, 2015; Li, Kong, Tan, & Wang, 2007). Agboola and Corey (1973) have also shown that soil organic matter shows great control over the concentration and release of major and minor nutrients, hence may be the reason N, P, and K showed very close relation to SOC in our study. Loss of these nutrients may reciprocally affect long‐term carbon sequestration. More especially, N‐loss in the long term may cause extensive carbon loss. Since N‐availability may enhance C sequestration (Deng, Wang, Liu, & Shangguan, 2016) through biomass growth, its limitation due to degradation may therefore significantly reduce C sequestration in degraded ecosystems in the long term.

Our results showed increase in SOC in HV and SD between 2013 and 2015 but a reduction in MD and HD in the same period. This may be as a result of long‐term effect of vegetation loss on SOC which arose due to exposure of the more degraded vegetation in MD and HD to further degradation processes in the long term. Schleuss et al. (2015) in their study of grassland degradation effects on SOC concluded that significant SOC loss due to degradation is as a result of lack of root C‐input, erosion and soil organic matter decomposition. Variations in SOC within the study years may also be attributed to variations in annual climatic conditions.

Soil organic carbon dynamics along the vertical profile showed a decreasing trend. More SOC were accumulated in the 0–10 cm profile than the deeper profiles. This is in line with other studies which indicated that top soils accumulate more carbon than deeper soil profiles (Deng et al., 2016), and this could be attributed to higher carbon input within the root zone through root biomass C allocation. In the current study, we found that vegetation degradation affected vertical distribution of SOC. Soil organic carbon in the 0–10 cm in HV and SD were 45.59% and 51.01% higher compared with their 20–40 cm profiles, respectively, but was only 25.26% and 16.88% higher in MD and HD, respectively. Higher moisture and root activity in the vegetated soils may have caused higher C allocation in the top soil, hence higher proportions of SOC in the top soil (0–10 cm) in HV and SD than in the less vegetated soils (MD and HD). However, environmental controls on SOC diminished along the soil profile. Table 4 showed significant relations of SOC with BD, SWC, and ST but with diminishing effect along soil depth. The environmental control of SOC being strongest in the top soil is consistent with reports by other researchers (Jobbágy & Jackson, 2000).

## 5 CONCLUSIONS

Our results indicated that in the wet meadows on the QTP, SOC reduced along the moisture gradient and with degree of wetland vegetation degradation within the 0–40 cm profile. Changes in above and below ground biomass, soil physical properties, and soil hydrothermal conditions had influence on soil carbon input. Soil organic carbon variations were closely related to TN, TP, and TK. Belowground biomass, AGB and SWC were the most important factors influencing SOC content which correlated positively with SOC while BD and ST correlated negatively with SOC. Approximately, 50% reduction of SOC content was observed in the HD wet meadows, compared to the healthy wet meadows, implying that under climate warming, vegetation, and soil moisture loss will dramatically destabilize carbon sink capacities of wetlands. These results on the changes in SOC along moisture and vegetation degradation gradient and their relationships will help deepen our understanding on internal and external controls of SOC under degraded wet meadow soils which will improve monitoring and restoration of the ecological health of ecosystems on the QTP and similar ecosystems.

## CONFLICT OF INTERESTS

None declared.

## AUTHOR CONTRIBUTIONS

Financial grants were awarded to M.W. and L.G.; J.Z., W.M. and G.L. conceived and designed the experiments; A.M.A. and J.W. performed the experiments; A.M.A. and G.C. analyzed the data; while A.M.A. wrote the paper with editing support from J.Z.

## DATA ACCESSIBILITY

The data used in this publication have been archived and published in figShare and the published DOI is https://doi.org/10.6084/m9.figshare.6297389.v1.
